# Remission of irritable bowel syndrome with lifestyle medicine

**DOI:** 10.12669/pjms.41.7.12120

**Published:** 2025-07

**Authors:** Palwasha Shabbir, Samina Malik, Manal Naveed, Muhammad Farhan Anwar, Mateen Sheraz, Hassan Zahid Waraich

**Affiliations:** 1 Palwasha Shabbir (PS), University College of Medicine and Dentistry (UCMD), The University of Lahore (UOL), Lahore, Pakistan; 2 Samina Malik (SM) , University College of Medicine and Dentistry (UCMD), The University of Lahore (UOL), Lahore, Pakistan; 3 Manal Naveed (MN) , University College of Medicine and Dentistry (UCMD), The University of Lahore (UOL), Lahore, Pakistan; 4 Muhammad Farhan Anwar (MFA) University College of Medicine and Dentistry (UCMD), The University of Lahore (UOL), Lahore, Pakistan; 5 Mateen Sheraz (MS) , University College of Medicine and Dentistry (UCMD), The University of Lahore (UOL), Lahore, Pakistan; 6 Hassan Zahid Waraich (HZW) , University College of Medicine and Dentistry (UCMD), The University of Lahore (UOL), Lahore, Pakistan

**Keywords:** Gut-Brain Axis, Irritable Bowel Syndrome (IBS), Low FODMAP Diet, Lifestyle Medicine, Physical Activity, stress management, sleep optimization

## Abstract

Irritable bowel syndrome (IBS) is a chronic symptom complex diagnosed by the ROME-IV criteria with a global incidence of 7-18%. Pharmacological treatment is largely symptomatic, relying on laxatives and neuromodulators. Previous studies have linked IBS to individual factors such as stress, poor sleep, lack of dietary discipline and physical activity. However, data on remission through a comprehensive lifestyle medicine approach remains limited. This intervention included 12 week period of structured sleep routine. It included compliance with a nocturnal sleep schedule from 10-11 pm (bedtime) to 7 am, reading before bedtime, limiting screen time and caffeine consumption especially in late evening. The intervention at the level of stress management involved counseling by a psychiatrist and a nutritionist and mindfulness-meditation. The intervention at the level of physical activity included tracking steps for at least 45 minutes per day using a diary. Personal weekly zoom meetings were conducted for each participant by research team members to ensure thorough understanding of compliance diary. This was in addition to consultation with a nutritionist and a psychiatrist. Compliance was monitored and tailored guidance was provided. To assess comprehension, participants were asked to summarize the instructions provided during the sessions. They were subsequently able to explain the contents and structure of their compliance diaries in a clearly and consistently manner. These efforts ensured a standardized lifestyle management over 12 weeks period. Dietary management included self-documented adherence to a low FODMAP diet after providing them with a list of high versus low FODMAP diet. Participants demonstrated notable improvements in sleep quality, a decrease in stress levels, physical activity, and adherence to a low FODMAP diet which was significantly associated with remission of symptoms. A limitation of the study was small sample size (n=6) as patients became increasingly difficult to follow up with due to multiple dropouts (attrition) during the study. These constraints reduced the power and generalizability of the results. Larger and long-term studies are needed to confirm these findings.

Irritable Bowel Syndrome (IBS) is a chronic functional gastrointestinal disorder characterized by abdominal discomfort, bloating, and a change in bowel habits. It affects almost 11% of the global population, while Pakistan has an incidence of 33%^1^. It appears to be more common in medical-undergraduates due to factors like constant stress^2^, poor sleep quality,^3^ and reduced physical activity^4^ along with compromised diet quality^5^ due to academic workload. Stress may worsen IBS by disturbing the gut-brain-axis, changing motility, and increasing the sensitivity of the intestine^6^. Furthermore, decreased sleep quality may reduce immunity and cause fatigue, mood swings, and anxiety^2^. Likewise, dietary discipline^5^ plays a key role in controlling IBS. To reduce IBS symptoms, a low FODMAP (fermentable oligosaccharides, disaccharides, monosaccharides, and polyols) is recommended. The high FODMAP diet includes short-chain carbohydrates, or sugars, which cause indigestion in IBS patients due to poor absorption from small intestine^5^. Similarly, physical activity has been recognized for managing chronic stress^2^. However, the long-term effects of combined lifestyle medicine remain ambiguous. Also, dysregulation of gut-brain axis, visceral hypersensitivity, altering intestinal microbiota, and immune activation are described in pathophysiology of IBS^6^.

The usual classic pharmacological therapy approach, such as antispasmodics, laxatives, and neuromodulators, only targets the symptoms rather than treating the underlying mechanism. A recent case-study reinforces the role of lifestyle medicine in regulating gut physiology and microbiota composition by focus on physiology and highlights stress, poor sleep quality, inflammation-inducing diet, and a sedentary lifestyle as common aggressors to IBS symptoms.^7^ The current case-series observed the effects of lifestyle medicine intervention for 12 weeks. Regular weekly follow-up was conducted to check the compliance. Participants were instructed to maintain a diary to avoid forgetfulness along with mention of remission of symptoms. A total of six medical students from University College of Medicine and Dentistry, were inducted after getting diagnosed with IBS on ROME IV criteria by a gastroenterologist.^8^ This criterion includes recurrent abdominal pain that is associated with defecation or altered bowel habits (that is: constipation, diarrhea or a mix of constipation and diarrhea). Patients may also experience a bloating state. Symptoms must have first appeared at least six months prior to diagnosis and remained present over the past three months. Their ethical approval was obtained (ERC/28/23/02) from UCMD’s ethical committee. The purpose was to understand the association of modifiable lifestyle factors with remission and exacerbation of the disease symptoms.

## Inclusion and Exclusion Criteria:

The medical undergraduates diagnosed with IBS symptoms based on ROME-IV^8^ criteria for more than six months with documentary evidence were included upon informed consent. The students suffering from organic GI disorders, recent antibiotic use, and chronic inflammatory diseases were excluded. The study was conducted at UCMD from 2023-2024 (June 2023 to August 2024). The participants reported a significant improvement after intervention in sleep quality (p = 0.024), on stress reduction (p = 0.013), on physical activity (p = 0.005), and on adherence to a low FODMAP diet (Table-I). This suggests that besides pharmaceutical treatment, a broad lifestyle modification may influence the gut-brain connection, microbial diversity, and remission of symptoms. This can be further validated by randomized controlled trials. The intervention significantly reduced IBS discomfort, improved sleep quality, and reduced gastrointestinal distress.

**Table-I:** Lifestyle Intervention Before and After with low FODMAP diet Frequency.

Variables	Before Lifestyle Intervention Mean ±SD	After Lifestyle Intervention Mean ±SD	P-Value (≤0.05)
Sleep quality	9.16±5.00	1.79±0.96	0.023
Stress Levels	49.50±14.72	17.66±9.26	0.012
Physical Activity	22.66±11.25	45.16±11.92	0.005
***Frequency of Low FODMAP Diet Consumption Before and After the Intervention***			
0-2 times/week	66.667%	0 %	
3-4 times/week	33.3 %	66.667%	
>5 times/week	0 %	33.3 %	

***Note:*** See text for interpretation.

The percentage of participants adhering to the Low FODMAP diet, was assessed on weekly basis, using a food frequency questionnaire.^5^ Sleep quality was evaluated through the Pittsburgh Sleep Quality Index^3^ where a global score of 0–4 indicated good sleep quality, while scores between 5–21 reflected sleep disturbances. Visceral sensitivity was measured using the Visceral Sensitivity Index^2^ categorizing participants into low (0-29), moderate (30-55), or high (56 and above) visceral sensitivity. Physical activity levels were determined using the Godin-Shephard Leisure-Time Physical Activity Questionnaire^4^, classifying individuals as insufficiently active (score 1-14), moderately active (score 14-23), or active (score >24), providing a comprehensive assessment of lifestyle factors influencing IBS remission. Regular exercise and a low FODMAP diet appeared to improve their overall health. Their perceived intestinal health was reported to be improved by lifestyle changes. Statistical analyses were performed using paired t-test to assess pre- and post-intervention differences (p < 0.05 considered significant).

**Fig.1:**
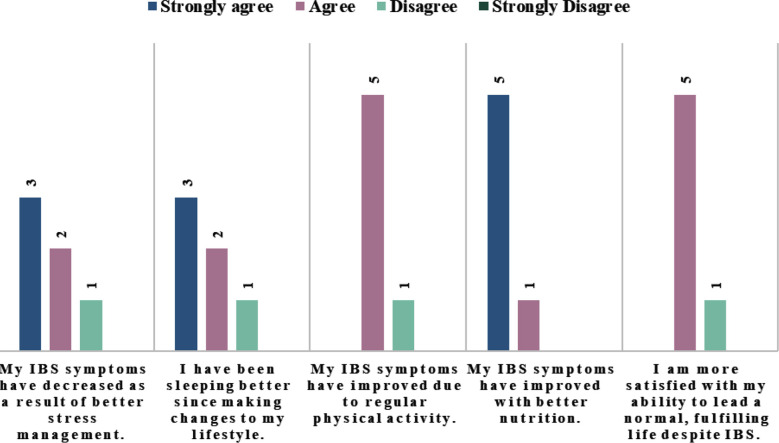
Impact of lifestyle changes on IBS symptoms.

As indicated in Table-I, before the intervention, adherence to the recommended low FODMAP diet was low. Table shows that 0% participants consumed low FODMAP diet, >5 times/week, and a majority (66.7%) reported the consumption of low FODMAP diet, 0-2 times/week. This implied limited or no adherence to the recommended low FODMAP diet. Following the intervention, adherence drastically improved. The 0% frequency (0-2 times/week) was completely eliminated, indicating all participants consumed the recommended low FODMAP diet to some degree and reduced high FODMAP diet consumption. Concurrently, 33% of participants achieved high adherence (>5 times/week), and a majority (66.6%) maintained consistent consumption (3-4 times/week), demonstrating a clear shift towards the recommended low FODMAP intake. See Fig.1 for impact of lifestyle changes on IBS symptoms.

The results suggested comprehensive lifestyle therapy as a potent non-pharmacologic method to treat IBS due to its multifactorial nature. Synergies between lifestyle elements and gut physiology were highlighted by improvement in stress, sleep, diet, and physical activity in the current study. Low FODMAP diet is likely to reduce bacterial agitation, bloating, and dysbiosis. Regular physical activity is recognized to increase gut motility and microbial diversity, possibly contributing to symptom relief. While the previous surveys were focused on cross-sectional individual or few lifestyle variables of the IBS research participants, this study combines several interventions with a follow-up of 12 weeks.

## Novelty Statement:

Previously, the corresponding author worked on a case-study with these lifestyle interventions^7^. However, this case-series offers a relatively longitudinal perspective with a 12-week follow-up on IBS using a comprehensive lifestyle technique. It integrates stress reduction, sleep optimization, physical activity, and low FODMAP diet intervention. This demonstrates demonstrates statistically significant reduction in symptoms complementing the pharmacologic treatment. To date, no prior study has holistically examined the combined impact of these key lifestyle parameters. The current case-series uniquely integrates all these lifestyle factors, offering a comprehensive perspective on their collective role in managing and potentially remitting IBS symptoms.

## Limitations:

The study was limited by a small sample size and a short follow-up period, attributed to the hectic routines. This resulted in gradual decline of cases, who were removed from the study respecting their request. Furthermore, funding was not available to test fecal and hematological markers etc.

## Future Directions:

Larger randomized control trials, gut biomarker examination, personalized nutrition, wearable health technologies and prognostic markers can be incorporated in future research.

## Ethical consideration:

The study was approved by the Ethical Review Board (ERC/28/23/02, dated February 8, 2023) of The University of Lahore. All participants provided written informed consent.

## References

[1] Zeeshan MH, Vakkalagadda NP, Sree GS, Anne KK, Parkash O, Fawwad SB (2022). Irritable bowel syndrome in adults:Prevalence and risk factors. Ann Med Surg (Lond).

[2] Cojocariu R, Ciobica A, Balmus INM, Gorgan L, Padurariu M, Stanciu C (2019). Some mechanistical and computational aspects on the correlations that might exist between irritable bowel syndrome versus sleep patterns and disturbances. In:2019 E-Health and Bioengineering Conference (EHB).

[3] Moloney RD, Johnson AC, O'Mahony SM, Dinan TG, Greenwood-Van Meerveld B, Cryan JF (2016). Stress and the microbiota–gut–brain axis in visceral pain:relevance to irritable bowel syndrome. CNS Neurosci Ther.

[4] Godin G (2011). The Godin-Shephard leisure-time physical activity questionnaire. Health Fit J Canada.

[5] Cozma-Petruţ A, Loghin F, Miere D, Dumitraşcu DL (2017). Diet in irritable bowel syndrome:What to recommend, not what to forbid to patients!. World J Gastroenterol.

[6] Drossman DA, Hasler WL (2016). Rome IV—functional GI disorders:disorders of gut-brain interaction. Gastroenterology.

[7] Shujaat T, Malik S (2022). Development of a dietary and lifestyle algorithm for prophylactic-remission of IBS:a case study. FASEB J.

[8] Lacy BE, Patel NK (2017). Rome criteria and a diagnostic approach to irritable bowel syndrome. J Clin Med.

